# Analysis of spatial–temporal dynamic distribution and related factors of tuberculosis in China from 2008 to 2018

**DOI:** 10.1038/s41598-023-31430-0

**Published:** 2023-03-27

**Authors:** Mingjin Xue, Jinlin Zhong, Miao Gao, Rongling Pan, Yuqian Mo, Yudi Hu, Jinlin Du, Zhigang Huang

**Affiliations:** 1 School of Public Health, Guangdong Medical University, Dongguan, Guangdong Province China; 2 Pension Industry Research Institute, Guangdong Medical University, Dongguan, Guangdong Province China

**Keywords:** Epidemiology, Infectious diseases, Public health

## Abstract

Through spatial–temporal scanning statistics, the spatial–temporal dynamic distribution of pulmonary tuberculosis incidence in 31 provinces and autonomous regions of China from 2008 to 2018 is obtained, and the related factors of spatial–temporal aggregation of tuberculosis in China are analyzed to provide strong scientific basis and data support for the prevention and control of pulmonary tuberculosis. This is a retrospective study, using spatial epidemiological methods to reveal the spatial–temporal clustering distribution characteristics of China's tuberculosis epidemic from 2008 to 2018, in which cases data comes from the China Center for Disease Control and prevention. Office Excel is used for general statistical description, and the single factor correlation analysis adopts *χ*^2^ Test (or trend *χ*^2^ Inspection). Retrospective discrete Poisson distribution space time scanning statistics of SaTScan 9.6 software are used to analyze the space time dynamic distribution of tuberculosis incidence in 31 provinces, cities and autonomous regions in China from 2008 to 2018. ArcGIS 10.2 software is used to visualize the results. The global spatial autocorrelation analysis adopts Moran's I of ArcGIS Map(Monte Carlo randomization simulation times of 999) is used to analyze high-risk areas, low-risk areas and high-low risk areas. From 2008 to 2018, 10,295,212 cases of pulmonary tuberculosis were reported in China, with an average annual incidence rate of 69.29/100,000 (95% CI: (69.29 ± 9.16)/100,000). The annual GDP (gross domestic product) of each province and city showed an upward trend year by year, and the number of annual medical institutions in each province and city showed a sharp increase in 2009, and then tended to be stable; From 2008 to 2018, the national spatiotemporal scanning statistics scanned a total of 6 clusters, including 23 provinces and cities. The national high-low spatiotemporal scanning statistics of the number of pulmonary tuberculosis cases scanned a total of 2 high-risk and low-risk clusters. The high-risk cluster included 8 provinces and cities, and the low-risk cluster included 12 provinces and cities. The global autocorrelation Moran's I index of the incidence rate of pulmonary tuberculosis in all provinces and cities was greater than the expected value (E (I) = −0.0333); The correlation analysis between the average annual GDP and the number of pulmonary tuberculosis cases in each province and city from 2008 to 2018 was statistically significant. From 2008 to 2018, the spatial and temporal scanning and statistical scanning areas of tuberculosis incidence in China were mainly concentrated in the northwest and southern regions of China. There is an obvious positive spatial correlation between the annual GDP distribution of each province and city, and the aggregation degree of the development level of each province and city is increasing year by year. There is a correlation between the average annual GDP of each province and the number of tuberculosis cases in the cluster area. There is no correlation between the number of medical institutions set up in each province and city and the number of pulmonary tuberculosis cases.

## Introduction

Tuberculosis is a chronic infectious disease caused by *Mycobacterium tuberculosis* and one of the top ten causes of death in the world. According to the global tuberculosis report 2022, the number of new tuberculosis patients in China ranked sixth among the 30 high burden countries in 2021, making China one of the world's top ten high burden countries for tuberculosis^1^. The report of the fifth national tuberculosis epidemiological sampling survey in China shows that^2^: The incidence of pulmonary tuberculosis in China has a typical regional distribution feature of high in the west and low in the east. There is a large difference between different regions, and the epidemic situation in some regions is still very serious.

Studies have shown that the incidence of pulmonary tuberculosis has spatial aggregation^3,4^. Spatial epidemiology is a new branch of epidemiology, which is a discipline to describe and analyze the geographical distribution of diseases. It mainly applies geographic visualization technology to provide intuitive distribution of diseases and research on spatial distribution patterns. Spatial epidemiology can analyze the geographical analysis of diseases in the study area, so as to obtain the scope and changes of disease aggregation, which plays a key role in health decision-making^5^. However, up to now, few research results have been reported on tuberculosis in terms of economic and medical security.

This study intends to use the spatial–temporal scanning statistical method of spatial epidemiology to reveal the spatial–temporal clustering distribution characteristics of the tuberculosis epidemic, and analyze the high and low risk clustering regions of the annual reported case data of tuberculosis in 31 provinces and cities in China (excluding Hong Kong, Macao and Taiwan, because the data in these three places shows no data) from 2008 to 2018. To analyze the impact of the annual incidence rate of tuberculosis, the number of medical institutions in each province and city, and the gross domestic product (GDP) of each province and city on the space–time distribution of tuberculosis. The purpose is to provide a reference basis for formulating or improving current tuberculosis prevention and control strategies and measures.

## Materials and methods

### Data source

China's TB case report data from 2008 to 2018 comes from the infectious disease information management system of the public health scientific data center set up by the China Center for Disease Control and prevention. According to the annual data conditions of each province and city, download the annual TB infectious disease report data of each province and city; The annual number of medical institutions in each province and city, the population data of each province and city, and the annual gross domestic product (GDP) of each province and city are all derived from the statistical yearbook of the China Bureau of Statistics.

### Method

This is a retrospective study, and the following methods are mainly applied.

1. Descriptive analysis: use Office Excel 2019 to establish case database, and use Office Excel 2019 software for general statistical description.
2. *χ*^2^ Inspection: SPSS 25.0* χ*^2^ Test and analyze the factors related to the aggregation of cases of tuberculosis, compare the correlation between the average annual GDP of each province and city, the average annual number of medical institutions and the aggregation area of cases of tuberculosis. The GDP correlation analysis is divided into low development economic areas and high development economic areas.

The comparison standard is: the average annual GDP of each province and city from 2008 to 2018 is compared with the average annual GDP of China from 2008 to 2018 (that is, the average total GDP from 2008 to 2018 divided by 31 provinces and cities in China), If it is lower, it is a low development economic zone; otherwise, it is a high development economic zone. Similarly, the relevant analysis of the number of medical institutions is divided into areas that meet the medical standard and areas that do not meet the medical standard. The comparison standard is: the average number of medical institutions in each province and city from 2008 to 2018 is compared with the average number of medical institutions in China from 2008 to 2018 (that is, the average number of total medical institutions in China from 2008 to 2018 divided by 31 provinces and cities in China), If it is lower than the standard, it is the area with unsatisfied medical level, otherwise it is the area with satisfied medical level.

Trend* χ*^2^ test the trend analysis of China's TB incidence rate data from 2008 to 2018, and the test levels were all taken as α = 0.05, *P* < 0.05 indicates that the difference is statistically significant. If the theoretical frequency is less than 5, the continuous correction formula shall be used for correction^6^.

3. The number of reported cases of pulmonary tuberculosis and their spatial and temporal distribution: Retrospective discrete Poisson distribution spatial and temporal scanning statistics of SaTScan 9.6 software are used to analyze the temporal and spatial dynamic distribution of pulmonary tuberculosis in 31 provinces, cities and autonomous regions (except Hong Kong, Macao and Taiwan) in 2008–2018, and ArcGIS 10.2 software is used to visualize the results^7^, set the scanning window to be cylindrical (the bottom area is the study area, and the height is the length of time), scan different times and areas with a dynamically changing scanning window, construct a test statistic log likelihood ratio (LLR) according to the actual and expected number of cases in and out of the scanning window to evaluate whether the number of cases in the window is abnormal, and calculate the relative risk (RR), accurately assess the risk of each cluster^8^.

The RR value is the largest and the difference is statistically significant, indicating that the risk of disease in this region is the largest, that is, this scanning window is a first level cluster, and other RR value windows with statistical significance are second, third, and fourth level clusters. Monte Carlo simulation is used to test whether LLR statistics are statistically significant. The number of simulations are equal to 999^9^. Based on the data fitting of the previous study, this study limited the maximum scanning radius to 30% of the total population at risk, the maximum scanning height to 30% of the total study period, and the scanning window is based on the time unit of year, with the best effect.

4. Global spatial autocorrelation analysis: the Moran's I analysis method in ArcGIS 10.2 is used to conduct global spatial autocorrelation analysis on the annual tuberculosis incidence rate, annual GDP and the number of medical institutions in various provinces and cities in China from 2008 to 2018. The value range of Moran's I is [−1, 1]. I > 0 indicates spatial positive correlation, and the closer the value is to 1, the higher the spatial aggregation; I < 0 indicates negative spatial correlation; I = 0 indicates that spatial aggregation is not significant^10^.
5. Inclusion and exclusion criteria: We selected 31 provinces in China, excluding Hong Kong, Macao and Taiwan. This is because the domestic data of these three regions are independent. The parameter setting in the space time scanning statistics method is 30%, which is the result of repeated debugging in the previous research.
6. Statement for Ethics approval and consent to participate: Statement that the raw data used in this study does not require any administrative privileges and all data has been anonymized before acquisition (no information about any human is involved). This study statement confirms that all methods were carried out in accordance with relevant guidelines and regulations in the declaration.

### Ethical approval and consent to participate

Statement that the raw data used in this study does not require any administrative privileges and all data has been anonymized before acquisition (no information about any human is involved). This study statement confirms that all methods were carried out in accordance with relevant guidelines and regulations in the declaration.

## Result

### Descriptive analysis

#### Basic information of incidence rate of tuberculosis Annual Report

**Figure 1:**
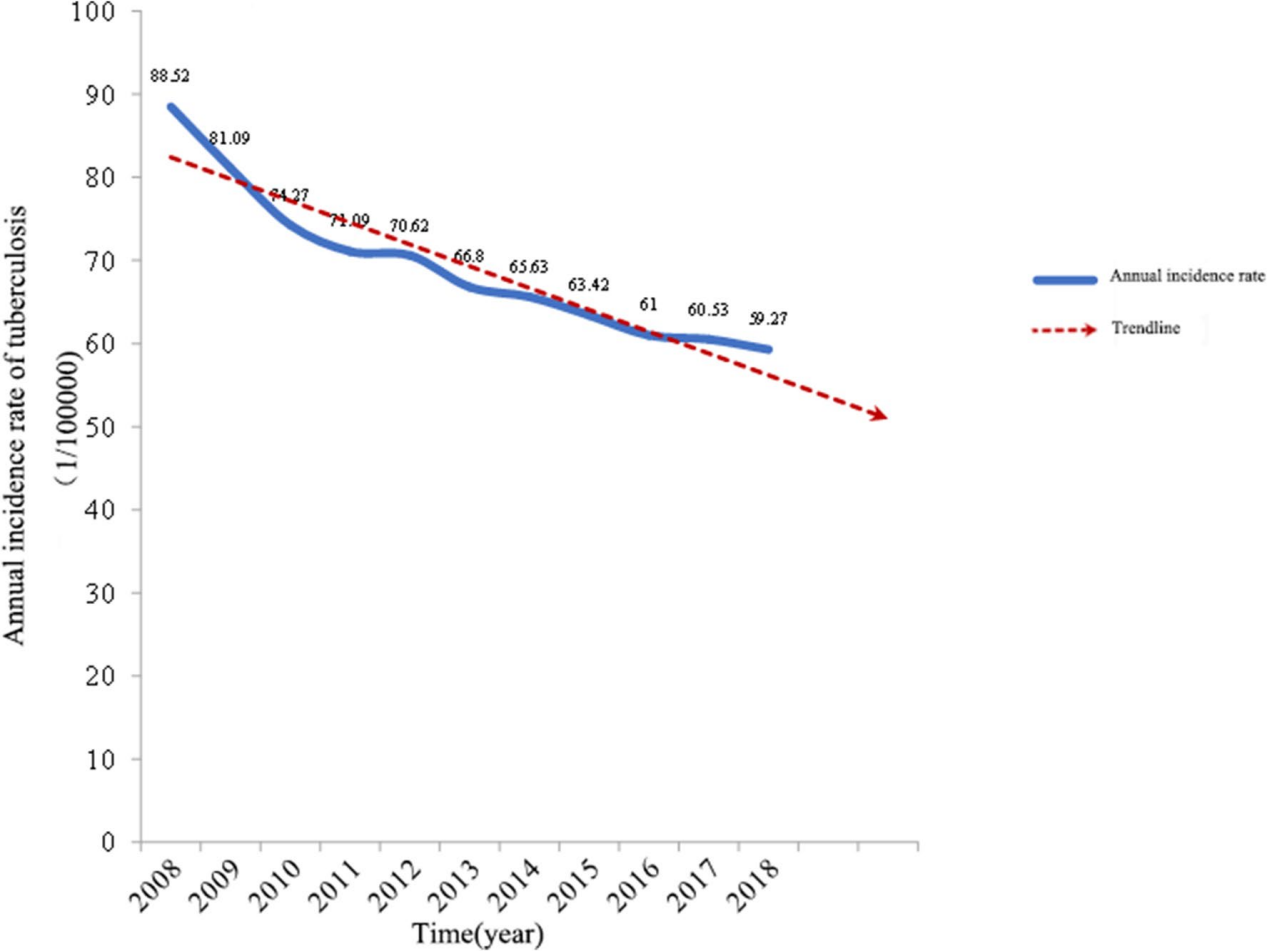
Statistics of annual incidence rate of tuberculosis in China from 2008 to 2018.

From 2008 to 2018, 10,295,212 cases of pulmonary tuberculosis were reported in China, with an average annual incidence rate of 69.29/100,000 (95% CI: (69.29 ± 9.16)/100,000). The annual reported incidence rate showed a downward trend year by year (trend *χ*^2^ = 687.506, *P* < 0.001) (Fig. 1 and Table 1).

**Table 1:** Reported incidence of pulmonary tuberculosis in China from 2008 to 2018.

Year	Cumulative number of cases	Reported incidence rate (per 100,000)	Mean value	95%CI	*χ*^2^	*P*
2008	1,169,540	88.52	69.29	(69.29 ± 9.16)	687.506	< 0.001
2009	1,076,938	81.09				
2010	991,350	74.27				
2011	953,275	71.09				
2012	951,508	70.62				
2013	904,434	66.80				
2014	889,381	65.63				
2015	864,051	63.42				
2016	836,236	61.00				
2017	835,193	60.53				
2018	823,342	59.27				

#### Number of medical institutions in various provinces and cities in China from 2008 to 2018 and annual GDP distribution

**Figure 2:**
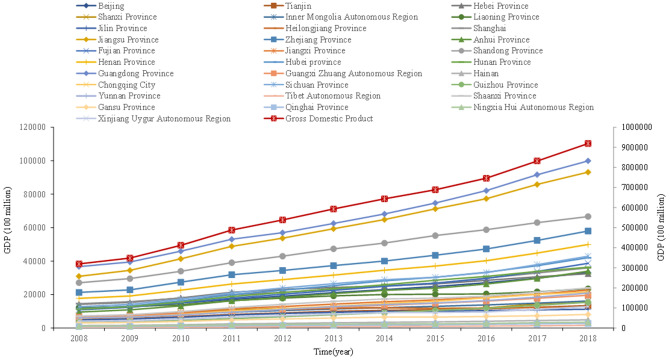
Annual GDP Changes of China's Provinces and Municipalities from 2008 to 2018.

GDP: From 2008 to 2018, the annual GDP of all provinces and cities in China showed an upward trend year by year, with Guangdong Province having the most obvious annual GDP change. The annual GDP of most northwest regions in China was at a low level (Fig. 2).

**Figure 3:**
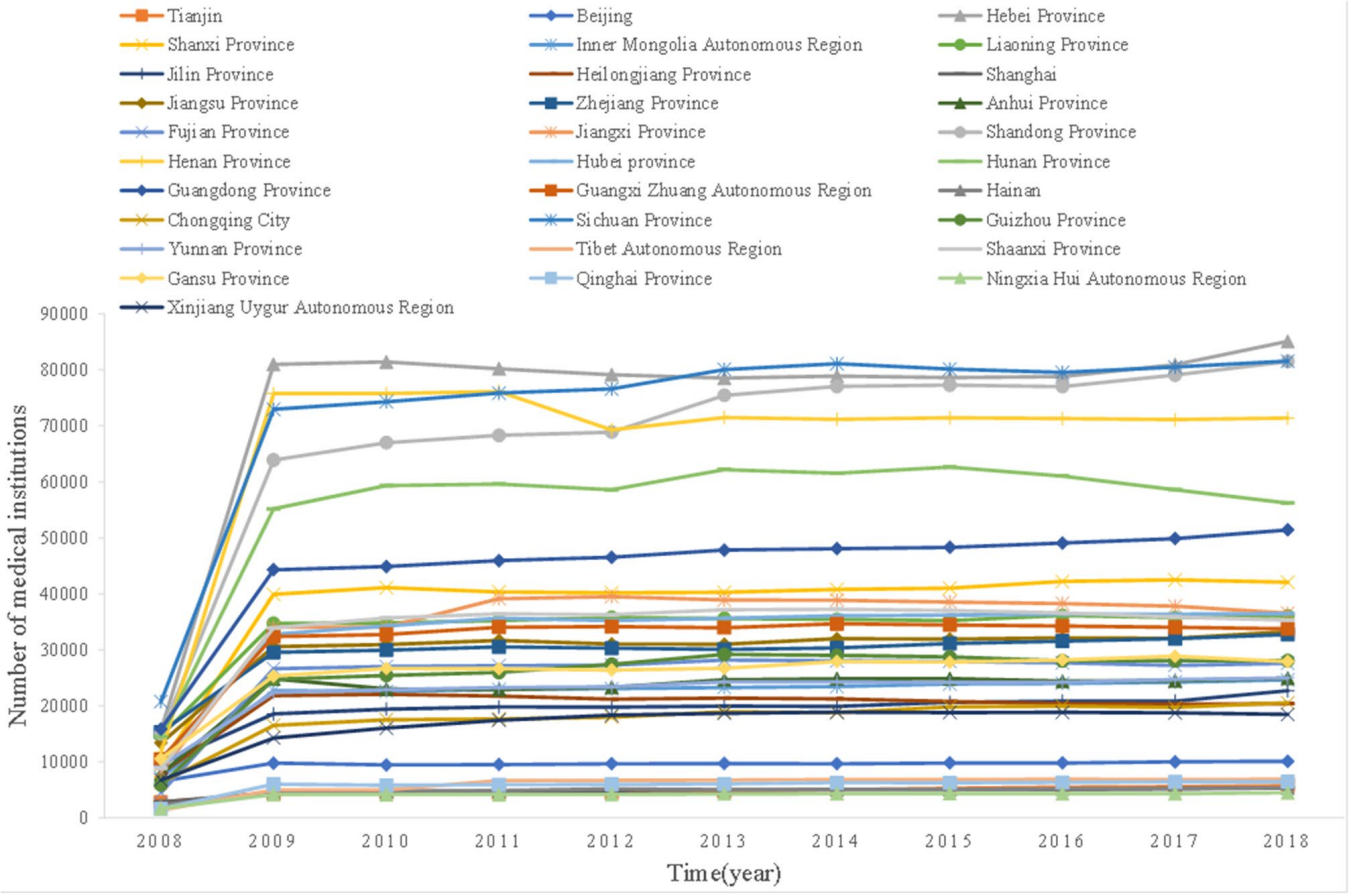
Changes in the number of medical institutions in China's provinces and cities from 2008 to 2018.

Number of medical institutions: from 2008 to 2018, the number of medical institutions set up in various provinces and cities in China showed a sharp increase in 2009, and then became stable. Among them, the number of medical institutions set up in Hebei Province increased most significantly, but the number of medical institutions set up each year in most regions of the country, such as the southwest, northwest, and central regions, has a low degree of change (Fig. 3).

#### Statistical analysis of the annual reported incidence of pulmonary tuberculosis by Space Time Scanning

From 2008 to 2018, a total of 6 clusters were scanned across the country, of which level 1 cluster included 3 provinces and cities, namely Xinjiang (RR = 3.28, 95%CI = 2.46 ~ 4.10), Qinghai (RR = 1.98, 95%CI = 1.49 ~ 2.48) and Tibet (RR = 2.17, 95%CI = 1.63 ~ 2.71). The clustering time was from 2016 to 2018, mainly in the western region of China (RR = 2.96, 95%CI = 2.22 ~ 3.70, LLR = 86,420.04, *P* < 0. 001). Level 2 cluster mainly includes five provinces and cities, namely Hainan (RR = 1.73, 95%CI = 1.30 ~ 2.16), Guangdong (RR = 0), Guangxi (RR = 0), Guizhou (RR = 2.47, 95%CI = 1.85 ~ 3.09) and Hunan (RR = 1.35, 95%CI = 1.01 ~ 1.69), which mainly occur in southwest China (RR = 1.65, 95%CI = 1.24 ~ 2.06, LLR = 85,178.82, *P* < 0. 001).

**Figure 4:**
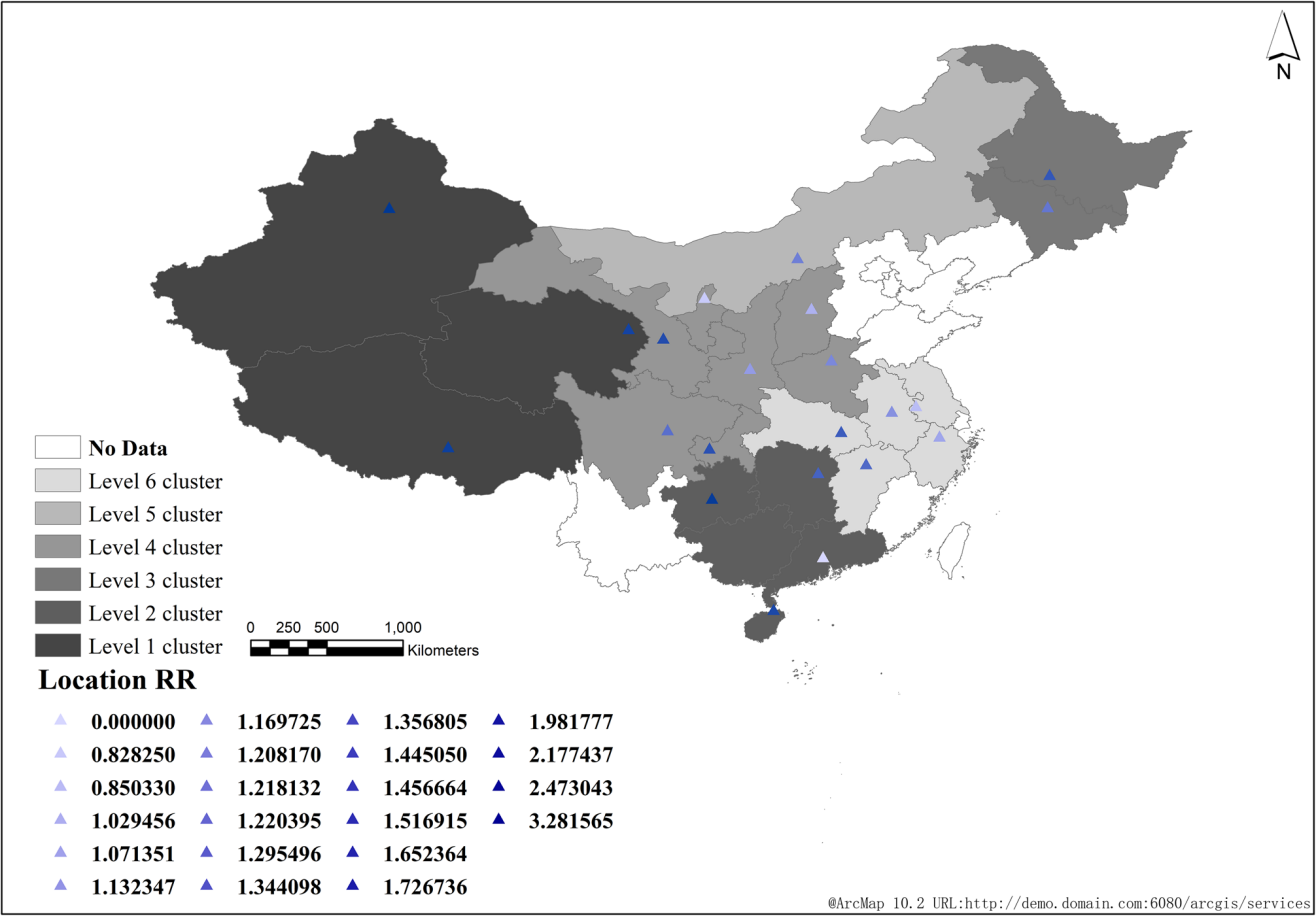
Space–time scanning analysis of reported cases of pulmonary tuberculosis in China from 2008 to 2018.

Level 3 cluster mainly includes two provinces and cities, Jilin (RR = 1.22, 95%CI = 1.01 ~ 1.53) and Heilongjiang (RR = 1.45, 95%CI = 1.09 ~ 1.81), which mainly occur in Northeast China (RR = 1.36, 95%CI = 1.02 ~ 1.70, LLR = 7758.50, *P* < 0.001). Level 4 cluster includes 7 provinces and cities (Shaanxi, Henan, Gansu, Shanxi, Ningxia, Chongqing, Sichuan, RR = 1.28, 95%CI = 1.10 ~ 1.60, LLR = 20,525.4, *P* < 0. 001), of which Gansu has the largest RR value (RR = 1.65, 95%CI = 1.04 ~ 2.77). Level 5 cluster only includes one province and city in Inner Mongolia (RR = 1.22, 95%CI = 1.01 ~ 1.62, LLR = 745.42, *P* < 0. 001). Level 6 cluster includes five provinces and cities, namely Anhui (RR = 1.17, 95%CI = 0.88 ~ 1.46), Jiangsu (RR = 0.85, 95%CI = 0.64 ~ 1.06), Hubei (RR = 1.44, 95%CI = 1.08 ~ 1.80), Zhejiang (RR = 1.07, 95%CI = 0.80 ~ 1.34) and Jiangxi (RR = 1.34, 95%CI = 1.00 ~ 1.68), which mainly occur in the middle and lower reaches of the Yangtze River in China (RR = 1.15, 95%CI = 1.08 ~ 1.44, LLR = 4257.15, *P* < 0. 001) (Table 2 and Fig. 4).

**Table 2:** Space time scanning analysis of reported cases of pulmonary tuberculosis in China from 2008 to 2018.

Cluster	Aggregation time(Year)	Gathering radius(km)	Number of provinces/cities	Number of observation cases	Expected number of cases	LLR	RR	95%CI	*P*
Level 1	2016–2018	1601.84	3	206,617	70,777.99	86,420.05	2.96	(2.22, 3.70)	< 0.001
Level 2	2008–2010	938.55	5	847,830	530,845.85	85,178.82	1.65	(1.24, 2.06)	< 0.001
Level 3	2008–2009	211.79	2	183,268	135,301.43	7758.50	1.36	(1.02, 1.70)	< 0.001
Level 4	2008–2010	608.53	7	796,718	634,535.84	20,525.48	1.28	(1.10, 1.60)	< 0.001
Level 5	2008–2009	0	1	40,994	33,677.18	745.42	1.22	(1.01, 1.62)	< 0.001
Level 6	2008–2009	373.60	5	461,180	402,504.94	4257.15	1.15	(1.08, 1.44)	< 0.001

**Figure 5:**
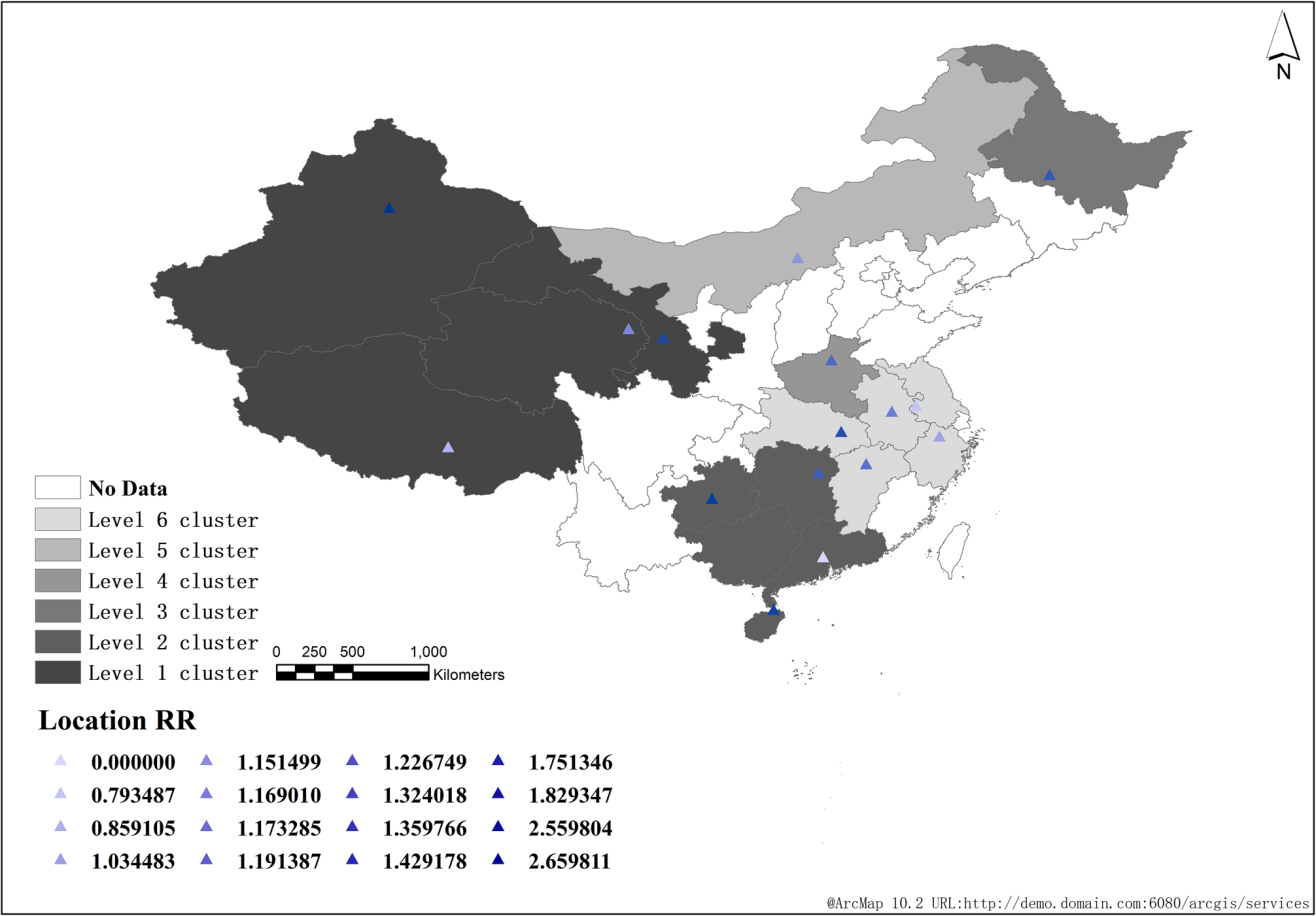
Space–time scanning analysis of reported cases of pulmonary tuberculosis in China from 2008 to 2013.

**Figure 6:**
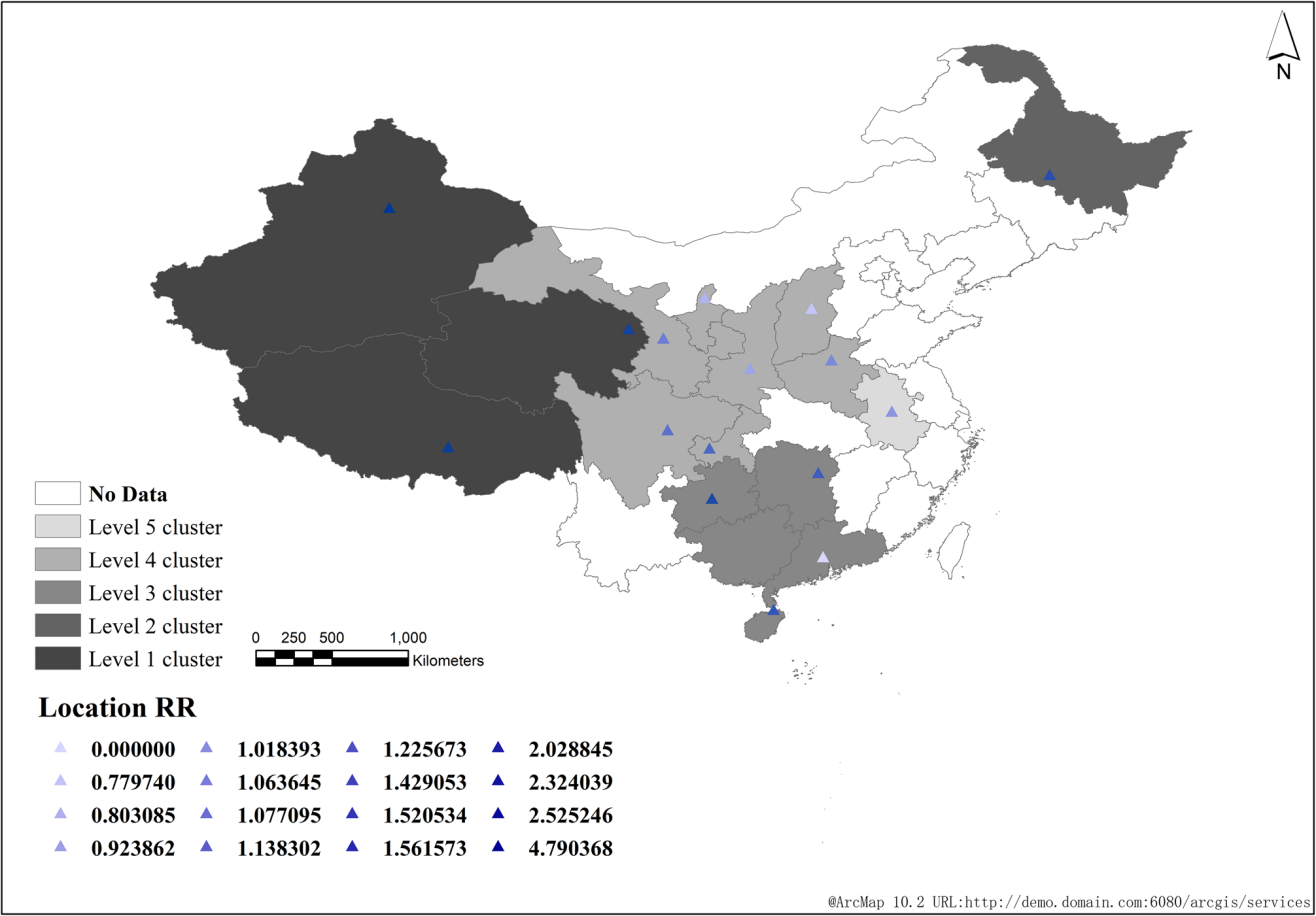
Space–time scanning analysis of reported cases of pulmonary tuberculosis in China from 2014 to 2018.

In the first six years, the average annual incidence rate in Northwest China (Xinjiang, Tibet, Qinghai and Gansu) was the highest, which was the first-class cluster (Fig. 5); In the last five years, the incidence of the disease increased and expanded to central China (Sichuan, Chongqing, Shaanxi, etc.) (Fig. 6). However, from 2008 to 2018, Xinjiang, Tibet and Qinghai had the highest incidence among all regions (Fig. 4).

### Global spatial autocorrelation analysis

#### Global self phase analysis of incidence rate of pulmonary tuberculosis

From 2008 to 2018, the global autocorrelation Moran's I index of the incidence rate of pulmonary tuberculosis in all provinces and cities in China was greater than the expected value (E (I) = −0.0333), of which the global Moran's I index was the smallest in 2018 (Moran's I = 0.2055), and the global Moran's I index was the largest in 2011 (Moran's I = 0.4126), and the p value was less than 0.001. This indicates that there is an obvious spatial positive correlation in the distribution of the national incidence rate of pulmonary tuberculosis. At the same time, the global Moran's I index showed a trend of rising first and then falling, indicating that the aggregation degree of its incidence rate gradually increased and then weakened (Table 3).

**Table 3:** Global spatial autocorrelation analysis of incidence rate of tuberculosis in China from 2008 to 2018.

Year	Moran’s *I*	Expected index	Variance	Z	*P*
2008	0.3262	−0.0333	0.0056	4.7806	< 0.001
2009	0.3672	−0.0333	0.0056	5.3498	< 0.001
2010	0.4058	−0.0333	0.0057	5.8170	< 0.001
2011	0.4126	−0.0333	0.0057	5.8645	< 0.001
2012	0.3620	−0.0333	0.0055	5.2841	< 0.001
2013	0.3671	−0.0333	0.0056	5.3312	< 0.001
2014	0.3628	−0.0333	0.0056	5.2934	< 0.001
2015	0.3396	−0.0333	0.0055	4.9869	< 0.001
2016	0.3135	−0.0333	0.0055	4.6662	< 0.001
2017	0.3109	−0.0333	0.0053	4.7086	< 0.001
2018	0.2055	−0.0333	0.0041	3.6898	< 0.001

#### Spatial autocorrelation analysis of annual GDP of provinces and cities in China

From 2008 to 2018, the overall autocorrelation Moran's I index of the annual GDP of all provinces and cities in China was greater than the expected value (E (I) = −0.0333), of which the overall Moran's I index was the smallest in 2008 (Moran's I = 0.1134), and the overall Moran's I index was the largest in 2018 (Moran's I = 0.1374), and the P value was less than 0.05, indicating that the annual GDP distribution of all provinces and cities in China has obvious positive spatial correlation. At the same time, the overall Moran's I index shows an upward trend, indicating that the concentration of development levels in various provinces and cities is increasing year by year(Table 4).

**Table 4:** Global Spatial Autocorrelation Analysis of GDP of China's Provinces and Municipalities from 2008 to 2018.

Year	Moran’s *I*	Expected index	Variance	Z	*P*
2008	0.1134	−0.0333	0.0056	1.9614	0.0498
2009	0.1209	−0.0333	0.0056	2.0592	0.0394
2010	0.1261	−0.0333	0.0056	2.1265	0.0334
2011	0.1259	−0.0333	0.0056	2.1227	0.0337
2012	0.1254	−0.0333	0.0056	2.1123	0.0346
2013	0.1258	−0.0333	0.0056	2.1189	0.0340
2014	0.1269	−0.0333	0.0056	2.1353	0.0327
2015	0.1270	−0.0333	0.0056	2.1405	0.0323
2016	0.1304	−0.0333	0.0056	2.1878	0.0286
2017	0.1305	−0.0333	0.0055	2.1905	0.0284
2018	0.1374	−0.0333	0.0056	2.2799	0.0226

#### Statistical analysis of high-low space–time scanning in tuberculosis cases

From 2008 to 2018, the statistical analysis results of high low time–space scanning of the number of tuberculosis cases in China showed that there were two high and low risk clusters scanned. The high risk clusters included 8 provinces and cities (Xinjiang, Qinghai, Tibet, Hainan, Guangdong, Guangxi, Guizhou, Hunan), and the low risk clusters included 12 provinces and cities (Inner Mongolia, Shanxi, Hebei, Beijing, Tianjin, Ningxia, Shandong, Shanghai, Zhejiang, Jiangsu, Anhui, Fujian).

The first level cluster of high risk is concentrated in northwest China, including 3 provinces and cities (Xinjiang, Qinghai, Tibet), and the second level cluster is concentrated in southern China, including 5 provinces and cities (Hainan, Guangdong, Guangxi, Guizhou, Hunan).

**Figure 7:**
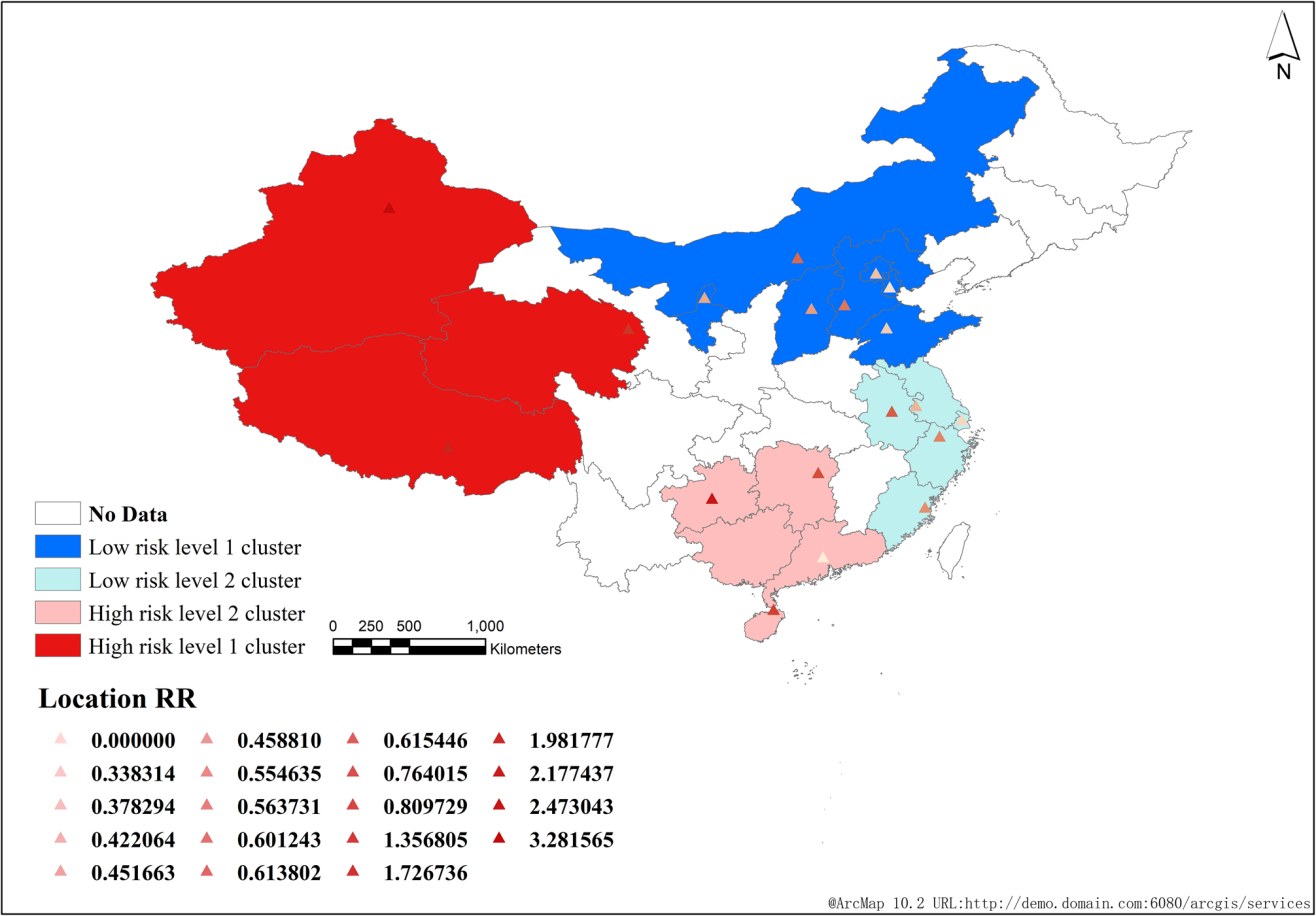
High-low space time scanning statistical analysis of reported cases of pulmonary tuberculosis in China from 2008 to 2018.

The first level cluster of low risk is concentrated in North China, including 7 provinces and cities (Inner Mongolia, Shanxi, Hebei, Beijing, Tianjin, Ningxia, Shandong), and the second level cluster is concentrated in the middle and lower reaches of the Yangtze River, including 5 provinces and cities (Shanghai, Zhejiang, Jiangsu, Anhui, Fujian) (Fig. 7).

### Cluster analysis of related factors in the number of cases of pulmonary tuberculosis

#### Correlation analysis between the average annual GDP and the number of cases of tuberculosis in China's provinces and cities from 2008 to 2018

The results showed that the correlation analysis between the average annual GDP and the number of tuberculosis cases in each province and city from 2008 to 2018 was statistically significant,* χ *^2^ = 8.343, *P* < 0.05, that is, the higher or lower the average annual GDP of each province and city, the higher the risk of pulmonary tuberculosis cluster area (Table 5).

**Table 5:** Correlation Analysis of Annual GDP and Tuberculosis Incidence Clusters in Provinces and Cities from 2008 to 2018.

Cluster	Economic development level		*χ*^2^	*P*
	High economic	Low economic		
High risk level 1 cluster area	0	3	8.343	0.039
High risk level 2 cluster area	2	3		
Low risk level 1 cluster area	3	0		
Low risk level 2 cluster area	5	4		

The correlation analysis between the annual average number of medical institutions in each province and city from 2008 to 2018 and the cluster area of the number of cases of pulmonary tuberculosis shows that the correlation analysis between the annual average number of medical institutions in each province and city from 2008 to 2018 and the cluster area of the number of cases of pulmonary tuberculosis has no statistical significance, *χ*^2^ = 3.377, *P* = 0.337.

## Discussion

### Basic situation of annual reported incidence rate of pulmonary tuberculosis and analysis of medical institutions in all provinces and cities and annual GDP results

From 2008 to 2018, China reported 10,295,212 cases of pulmonary tuberculosis, with an average annual incidence rate of 69.29/100,000. The annual reported incidence rate showed a downward trend year by year, with an average annual decline of 2.66%. The annual GDP of all provinces and cities in China showed an upward trend year by year, of which the annual GDP of Guangdong Province had the most obvious change, and the annual GDP of most northwest regions in China was at a low level from 2008 to 2018.

The number of medical institutions set up in various provinces and cities in China from 2008 to 2018 showed a sharp increase in 2009, and then became stable. Among them, the number of medical institutions set up in Hebei Province increased most significantly, but the change degree of the number of medical institutions set up each year in most regions of the country, such as southwest, northwest and central regions, was low. This is due to the remarkable effect of various relevant policies implemented since China's reform and opening up, the continuous improvement of people's quality of life, the continuous enhancement of the national medical level, the improvement of people's health security, and the continuous optimization of measures to prevent and control tuberculosis^1^.

But up to now, tuberculosis is still one of the respiratory infectious diseases with high incidence rate in China and even in the world^11^, The burden of disease it brings is increasing. Moreover, in the face of the novel coronavirus pneumonia epidemic that is still attacking the world, the new incidence rate of tuberculosis has decreased, but the burden of disease it brings may increase for patients who originally suffered from tuberculosis. This paper does not make an in-depth study on the association between COVID-19 and tuberculosis, but some studies point out that patients suffering from tuberculosis are also infected with COVID-19, Its mortality rate is higher than that of any single disease. In addition, the disease burden caused by simultaneous disease is higher than that caused by single disease^12^.

### Analysis of time–space scanning results of annual reported incidence of pulmonary tuberculosis

For the discussion of the method, we limit the maximum scanning radius to 30% of the total number of people at risk, the maximum scanning height to 30% of the total study period, and the scanning window takes years as the time unit, so the model fitting effect is the best. This is because we conducted simulation tests on some data in the previous study. We tried to compare the risk population at 5%, 10%, 20%, 30%, 40% and 50% respectively, and found that the fitting effect of the model was the best at 30%. The maximum limit of scanning radius and the maximum scanning height are also obtained by the same method.

From 2008 to 2018, a total of 6 clusters were scanned in China, including 23 provinces and cities. The gathering areas are mainly concentrated in the northwest and south of China. This time, it is the northeast and central regions, which is the same as the results of other studies^4,13–15^. At the initial stage of the study, we suspected that the display of this agglomeration area was closely related to the regional economic development.

By collecting the GDP values of each region since 2008–2018, we mapped the provinces and cities included in the agglomeration area one by one, and analyzed whether the degree of aggregation was related to the economy by using the single factor analysis method. The results showed that the correlation analysis between the average annual GDP of each province and city in 2008–2018 and the number of tuberculosis cases in the cluster area was statistically significant,* χ*^2^ = 8.343, *P* < 0.05, that is, the higher or lower the average annual GDP of each province and city, the higher the risk of pulmonary tuberculosis cluster area (Table 5).

These shows that, from 2008 to 2018, there is a high correlation between the economic development of the regions where tuberculosis is concentrated in China and that of the regions. This also explains that in areas with high economic development level or backward economy, the incidence of pulmonary tuberculosis has high concentration and high risk; The phenomenon of low incidence density and low risk of pulmonary tuberculosis in areas with medium economic development is similar to other research results^16–18^.

We guess there are several reasons as follows. First of all, there are relatively many medical institutions set up in places with high economic development level, and naturally the medical level of the cities or regions where they are located is relatively high. Some patients with pulmonary tuberculosis in backward regions will choose to go to these developed cities for medical treatment and treatment, which is one of the main reasons for increasing the incidence density of pulmonary tuberculosis.

Secondly, in economically developed areas, there are many immigrants, most of whom are from backward areas. These immigrants may themselves be patients with tuberculosis or asymptomatic infected people, and they are found after migration, which is one of the reasons for the high incidence of tuberculosis in economically developed areas.

Third, as mentioned in the first two points, the tuberculosis patients in underdeveloped areas do not choose to go to economically developed areas but stay there, and the local medical level is relatively backward, which cannot well control the spread of the disease, making the incidence of tuberculosis increase, which is the main reason for the increase in the aggregation of tuberculosis in underdeveloped areas.

Fourth, compared with the living habits of economically developed areas, backward areas are more likely to contact with live capture and livestock, which is also one of the risk factors for more susceptible to tuberculosis infection. In addition, the residents' lives are relatively close to each other, which increases the incidence clustering of tuberculosis.

As a result, the research results show that the areas where the incidence of tuberculosis is concentrated are closely related to the level of regional economic development, so we associate the number of medical structures. However, this study found that the results of the correlation analysis between the annual average number of medical institutions in each province and city from 2008 to 2018 and the cluster area of the number of cases of pulmonary tuberculosis showed that the correlation analysis between the annual average number of medical institutions in each province and city from 2008 to 2018 and the cluster area of the number of cases of pulmonary tuberculosis had no statistical significance, which showed that the number of medical institutions in the region was more or less had no correlation with the clustering of the incidence of pulmonary tuberculosis.

We consider the following two reasons. Firstly, the number of medical institutions is related to the control of disease transmission, but not necessarily to the gathering; Secondly, according to the actual needs of each place, each local government will issue the expectation number of medical institutions every year or every cycle, which may have nothing to do with the incidence of tuberculosis, but is a necessary measure. So we guess that this is one of the reasons why there is no statistical significance.

## Limitations

This study uses spatial and spatiotemporal methods to describe and analyze the distribution and influencing factors of the number of reported cases of pulmonary tuberculosis in China from 2008 to 2018, which has certain disease prevention and control value for tuberculosis itself, but there are still some limitations.

Firstly, for the internal validity of the research results, we chose retrospective research at the initial stage of the research, and the causal relationship is not so obvious. The results and reasons of the study were not explored in depth, but possible relevant factors were taken into consideration according to the social nature of tuberculosis itself. This limitation is obvious.

In addition, for the extrapolation effectiveness of the research results, it is not difficult to see that we do find the correlation between tuberculosis and regional economic development from the results of this study. This result proves that our guess is correct. This shows that this research method can be applied to different diseases or populations.

Secondly, our study is a retrospective study, and there is a certain selection bias. There is a defect in the inclusion of new cases, which is the same as other studies^19^. This is also the biggest limitation of this study. Thirdly, while studying tuberculosis itself, we cannot consider the impact of the recent novel coronavirus epidemic, and there are certain limitations in predicting the aggregation of tuberculosis in the future. We will fully include this factor in the follow-up research for research and analysis.

Fourthly, in the course of the study, we only found that the aggregation of tuberculosis incidence is related to the regional economic development, but not other possible related influencing factors, such as population migration, preventive measures, treatment plans, climate and meteorology. This is the inadequacy of this study, which we will further study. After all, we found that both economically developed areas and underdeveloped areas have a high degree of convergence in our research.

In the above discussion, several reasons were explained, but we did not consider whether the probability of urban residents (economically developed areas) suffering from tuberculosis was statistically significant compared with residents in backward areas? This is an analysis not done in this study. Considering the central theme of the study, it is not included in the research content. The latter can refer to this content for in-depth research.

## Conclusion

We used spatial epidemiological methods to reveal the spatial–temporal clustering distribution characteristics of China's tuberculosis epidemic from 2008 to 2018.

The annual reported incidence rate of tuberculosis in China from 2008 to 2018 showed a downward trend year by year, the annual GDP of each province and city showed an upward trend year by year, and the number of medical institutions set up in each province and city showed a sharp increase in 2009, and then tended to be stable.

From 2008 to 2018, China's space–time scanning statistics scanned a total of six clusters. The clustering areas were mainly concentrated in the northwest and southern regions of China, followed by the northeast and central regions. There was a significant spatial positive correlation between the distribution of incidence rate of pulmonary tuberculosis in various provinces and cities, and its incidence rate clustering degree gradually increased and then weakened.

The annual GDP distribution of all provinces and cities across the country has obvious positive spatial correlation, and the agglomeration degree of the development level of each province and city has increased year by year. From 2008 to 2018, there was a correlation between the average annual GDP of each province and the cluster area of the number of cases of tuberculosis, and there was no correlation between the average annual number of medical institutions in each province and the cluster area of the number of cases of tuberculosis.

## Data Availability

The data is publicly available. The data will be sent to the corresponding author by email or obtained through the following website: https://www.phsciencedata.cn/Share/edtShareNew.jsp?id=39208.
